# Report of an Obstetrical Case

**Published:** 1851-10

**Authors:** 


*Report of an Obstetrical Case.* By C. R. Palmore, M. D.,—Mr. Editor — Supposing the recital of the following case, which occurred in my practice whilst connected with the "Obstetric Institute" in Philadelphia, will be acceptable to you, and interesting, if not instructive, to the readers of your valuable magazine, I have taken the liberty to transmit a condensed report of it from my case-book for publication. It involves a question in obstetricy which has always puzzled the young physician not a little, and which I hope this case will, at least, serve to direct the attention of practical physicians to the elucidation of the mystery with which teachers and books have surrounded it.

*Case.* — April 10, 1850. Mrs. M. G. was seized with labor pains this morning. The labor proceeded very well (the vertex presenting in the first position), till the head emerged from the vulva. At this time the child took several deep inspirations and cried lustily. I immediately perceived, however, the umbilical cord around its neck, and, on more minute observation, discovered it drawn *thrice* very closely. The pains were now very severe, of the expulsive kind, which caused the funis to become tighter at every effort. I endeavored to pull the cord down and pass it over the head, but soon found the attempt useless from its extreme tenseness. I next attempted to disengage and suffer it to pass over the shoulders as they descended. This was also of no avail, for I could scarcely insert one finger between the cord and neck, so closely was it fastened. My next duty, I conceived, was to suffer it to *remain*, or, in other words, trust to the *vis medicatrix naturæ.* This negative plan was as nugatory as the others had been unfortunate. The child, which before had cried, now ceased. Its face became at first dusky, then black, exhibiting plainly the compression of the jugular veins and the consequent stagnation of blood in the brain. Here, then, was no time to be lost. The only alternative at my command was to sever the funis. But here (I should not call it *a demon*) authority arose before me. Dewees, Ramsbotham, Meigs, Hodge, whose opinions we all recognize, passed in rapid review. I remembered only one *similar* case, that mentioned in Dr. Meigs' "Treatise on Obstetrics," page 295. That this was a similar case, I had no doubt. I resolved, therefore, to cut the cord, which having been done, the child, released from its halter, soon revived; its face gradually assumed its natural hue; its breathing again commenced, and the mother's heart was illumed afresh by its reawakened cry. A few bearing down pains soon sufficed to drive the child from the vulva. I now tied the cord. Nothing unusual occurred in the after treatment.

*Remarks.* — It will be noticed that I pursued the usual course of treatment laid down in the books, and found this routine practice totally unavailing. Since the occurrence of the above case, I have examined the subject pretty closely, have maturely considered the salient points of the practice, and have come to the conclusion that the treatment usually recommended is, at least, irrational, if not radically defective, and that it will not answer in practice.

Upon a superficial examination, I know some may say that the success of the above case was *post hoc*, and not *propter hoc.* But if such persons would examine attentively the details given, they could not, in my opinion, refrain from being convinced that it was *propter hoc.*

What are the dangers that are so particularly inculcated by teachers? There is only one, and, to my mind, this scarcely deserves the name of danger. I refer to the *supposed* probability of the child's dying from loss of blood. I say *supposed,* because I cannot conceive why this can take place, as the child's body presses the cord against the vulva, effectually serving the purpose of a ligature. And this actually occurred in the case I have just narrated. The loss of blood was scarcely appreciable. This is the most prominent, if not the only objection that can be urged against the treatment.

The advantages are numerous, and, in my opinion, insuperable. It saves the child from impending death, and it empties the *placenta* of its retained blood, thus allowing it to be more easily detached by the efforts of the uterus, or, if necessary, by the hand. Moreover, if the cord were suffered to *remain* around the neck, and the child to descend, the cord might be torn from the *placenta* by the roots, or else it might draw the *fundus* of the uterus after it, and thus cause *inversio uteri.*

Those who have had this latter affection to deal with, will easily appreciate any plan recommended for its partial prevention. — *Stethoscope and Virginia Med. Gazette.*

